# Blue Light Acclimation Reduces the Photoinhibition of *Phalaenopsis aphrodite* (Moth Orchid)

**DOI:** 10.3390/ijms21176167

**Published:** 2020-08-26

**Authors:** Swee-Suak Ko, Chung-Min Jhong, Ming-Che Shih

**Affiliations:** 1 Academia Sinica Biotechnology Center in Southern Taiwan, Tainan 741, Taiwan; amo2175111@gmail.com; 2 Agricultural Biotechnology Research Center, Academia Sinica, Taipei 115, Taiwan; mcshih@gate.sinica.edu.tw

**Keywords:** high light stress, photoinhibition, blue light acclimation, photosynthesis, ETR, ROS

## Abstract

The moth orchid is an important ornamental crop. It is very sensitive to high light irradiation due to photoinhibition. In this study, young orchid tissue culture seedlings and 2.5” potted plants pretreated under blue light (BL, λ_max_ = 450 nm) at 100 µmol m^−2^ s^−1^ for 12 days (BL acclimation) were found to have an increased tolerance to high light irradiation. After BL acclimation, orchids had an increased anthocyanin accumulation, enhanced chloroplast avoidance, and increased chlorophyll fluorescence capacity whenever they were exposed to high light of 1000 μmol m^−2^ s^−1^ for two weeks (HL). They had higher Fv/Fm, electron transport rate (ETR), chlorophyll content, catalase activity and sucrose content when compared to the control without BL acclimation. Quantitative reverse transcription polymerase chain reaction (qRT-PCR) showed that transcript levels of *phototropins*, *D1*, *RbcS*, *PEPCK*, *Catalase* and *SUT2* were upregulated in the BL-acclimated orchids. Consequently, BL acclimation orchids had better growth when compared to the control under long-term high light stress. In summary, this study provides a solution, i.e., BL acclimation, to reduce moth orchid photoinhibition and enhance growth before transplantation of the young tissue culture seedlings and potted plants into greenhouses, where they usually suffer from a high light fluctuation problem.

## 1. Introduction

Photosynthesis converts light energy into chemical energy in photosynthetic plants and assimilates CO_2_ into carbohydrates. An increasing photosynthesis rate provides a way to increase plant growth and crop yield [1,2]. Photosystems (PS) consist of PSI and PSII. They are located in the thylakoid membranes and are the functional units for photosynthesis. PSII is very sensitive to high light-induced oxidative inactivation. Under high irradiation, the electron transport PSII is inhibited and its protein structure is damaged. In turn, PSI can be damaged if the electron transport from PSII is limited [3]. Photoinhibition reduces the quantum yield and light-saturated photosynthetic rate (A_max_) [4]. However, plants have developed several strategies to cope with photoinhibition. They turn on photoprotection mechanisms, including the regulation of light absorption and dissipation of excess light energy. Plants may change their leaf area, leaf angle and chloroplast movement, and may reduce their LHC antenna size. Once excess light has been absorbed, the plant will dissipate excess excitation energy through thermal dissipation [3,4].

Under high light conditions, chloroplasts can move away from the light (chloroplast avoidance) to reduce the antenna size, decrease light energy absorption and reduce photodamage. Phototropins play a key role in the chloroplast movement [5]. A recent study indicated that blue light induces chloroplast movement in several monocot plants such as *Brachypodium*, rye, wheat and barley [6], and *Phalaenopsis aphrodite* [7]. If excess light absorption cannot be avoided, the over-excitation of the light-harvesting complex pigments can be alleviated via the dissipation of excess light energy as heat via nonphotochemical quenching (NPQ) and manipulating the photosynthetic efficiency to enhance plant growth and significantly increase crop yield, as has been reported [8,9,10]. Plants also induce a photoprotective mode known as non-photochemical quenching of fluorescence (qN) and photochemical quenching of fluorescence (qP), for which a measurement in the dark is used to monitor the state of active PSII, enabling the detection of the early signs of photoinhibition [11]. When high irradiation produces excess protons, plants suppress photodamage by quenching heat. ROS comprises molecules such as superoxide (O_2_^−^), singlet oxygen (^1^O_2_^*^), hydrogen peroxide (H_2_O_2_) and hydroxyl radical (^•^OH). Singlet oxygen (^1^O_2_^*^) is formed mostly in the light in thylakoid membranes by PSII. It easily oxidizes biological molecules and is cytotoxic to plants. The ^1^O_2_^*^ production in chloroplasts can activate acclimation to high light or cell death [12]. In the meantime, plants will turn on reactive oxygen species (ROS) scavenging systems, such as superoxide dismutase (SOD), catalase (CAT) and ascorbate peroxidase (APX), to remove toxic ROS molecules [13].

Different plant species (shade- or sun-loving plants) may acquire photo-acclimation after exposure to a specific light quality and quantity [14]. Research compared shade-leaves, sun-leaves and leaves transferred from shade to full sunlight in begonia (a shade-loving plant) and indicated that transferred-leaves had low ETR, qP and NPQ values, and increased H_2_O_2_ and lipid hyperoxide accumulation under high light conditions, indicating that they did not acclimate completely [15]. It has been demonstrated that blue light (BL) is crucial for triggering photo-acclimation to high irradiance in plants [16,17] and diatoms [18]. It has been reported that the PSII unit size is larger when ferns are grown under BL [14]. Photo-acclimation of plants to high light increases the resistance of PSI to photoinhibition [19].

Early light-induced proteins (ELIPs) are light stress-induced and related to light-harvesting chlorophyll (LHC) a/b-binding proteins. ELIP1 increases with increasing high light intensities, and correlates with the degree of PSII photodamage [20]. NPQ alleviates photodamage by reducing ROS production [21]. The intrinsic reaction center protein (D1) is a target of photodamage. Photosystem II (PSII) photodamage is associated with the degradation and new synthesis of D1 [22]. Proteases of FtsH and Deg play predominant roles in D1 protein degradation [23,24]. Clearly, D1 protein turnover is important in protecting plants against photoinhibition.

*Phalaenopsis aphrodite* (moth orchid) is an important ornamental crop in the international trade market. The moth orchid performs a CAM mode photosynthesis and is a shade-loving ornamental plant. Depending on the cultivar, the photosynthesis rate of *Phalaenopsis* orchids peaks from a PPFD of 130 to 200 µmol m^−2^ s^−1^ [25,26]. Exposure to light higher than 325 µmol m^−2^ s^−1^ causes a significant photoinhibition of PSII [25]. However, *Phalaenopsis* plants are usually grown in a shade curtain greenhouse environment, which still has a high light above 300 µmol m^−2^ s^−1^, implying that orchids might suffer from a photoinhibition problem. Recently, BL receptors, phototropin 1 and 2 have been identified, and we found that at a BL greater than 25 µmol m^−2^ s^−1^ orchids started sensing high light and exhibiting chloroplast avoidance, and a significant chloroplast avoidance movement was observed at a BL greater than 100 μmol.m^−2^.s^−1^ [7]. Clearly, the moth orchid is very sensitive to high irradiance. Considering the significant economic impact of moth orchids, it is essential to eliminate photoinhibition and improve photosynthesis. So far, the photo-acclimation of orchids has not been studied. The objectives of this study were to compare the response of *Phalaenopsis* young seedlings and mature plants to moderate and high light stresses. We investigated whether BL pretreatment can induce light acclimation and whether BL acclimation enhances the photosynthesis efficiency of *Phalaenopsis*. Moreover, the underlying mechanisms of physiological, biochemical and molecular changes were investigated.

## 2. Results

### 2.1. Effect of Light Intensities on Orchid Chlorophyll Fluorescence Parameters

**Figure 1:**
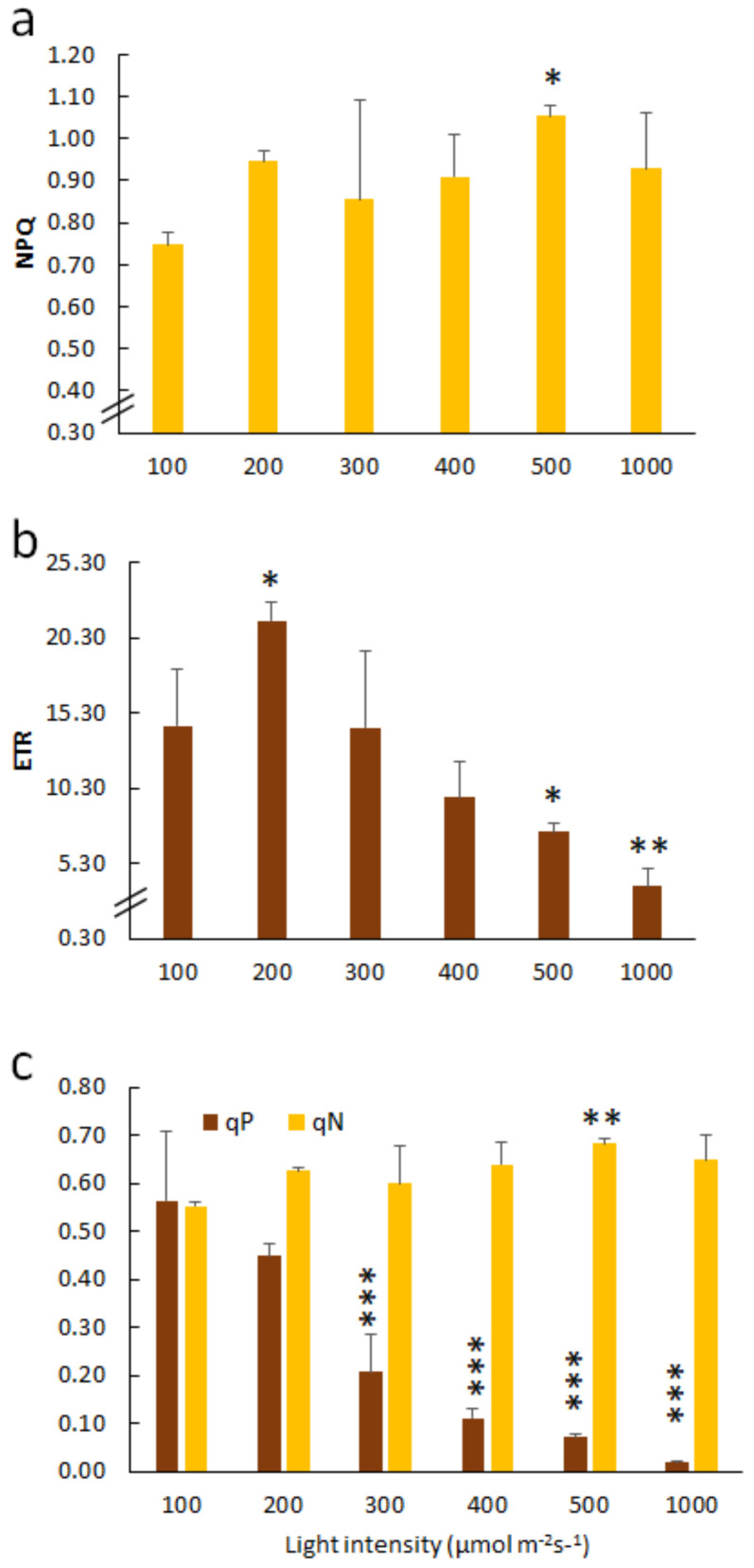
Light response curves of the chlorophyll fluorescence parameters of the moth orchid. (**a**) Non-photochemical quenching (NPQ), (**b**) electron transport rate (ETR), (**c**) photochemical quenching of fluorescence (qP) and non-photochemical quenching of fluorescence (qN) of orchids. Young seedlings were used in this experiment. A significant difference when compared to the “100 µmol m−^2^ s^−1^” is indicated with an asterisk. Statistical significance was determined by a one-way analysis of variance (ANOVA), followed by a Dunnett’s test. *, *p* < 0.05; **, *p* < 0.01; ***, *p* < 0.001. Error bars represent the standard error of the mean (*n* = 3).

To know how orchids respond to different light intensities and photoinhibition, we measured the chlorophyll fluorescence, under various light intensities from 100 to 1000 µmol m^−2^ s^−1^, of the young leaves of tissue culture seedlings that had been transplanted to a sphagnum moss pot for one month. The lowest non-photochemical quenching (NPQ) level was 100 µmol m^−2^ s^−1^, but the highest was 500 µmol m^−2^ s^−1^ (Figure 1a). The electron transport rate (ETRII) peaked at 200 µmol m^−2^ s^−1^ but decreased significantly at >500 µmol m^−2^ s^−1^ and decreased significantly to a negligible amount at 1000 µmol m^−2^ s^−1^ (Figure 1b). The level of qP was significantly decreased when the orchids were irradiated at ≥300 µmol m^−2^ s^−1^ and dropped to a negligible amount at 1000 µmol m^−2^ s^−1^ (Figure 1c). Compared to qP, the qN values did not alter much in the leaves of orchids responding to various light intensities (Figure 1c). These data indicated that when the moth orchid is exposed to high irradiation, it does not turn on the non-photochemical quenching mechanism, but the ETR and the oxidation state of the photosystem II (qP) are the most sensitive parameters in the moth orchid in response to light stress.

### 2.2. Blue Light Acclimation Enhanced Chlorophyll Fluorescence Capacity after Exposure to High Light

**Figure 2:**
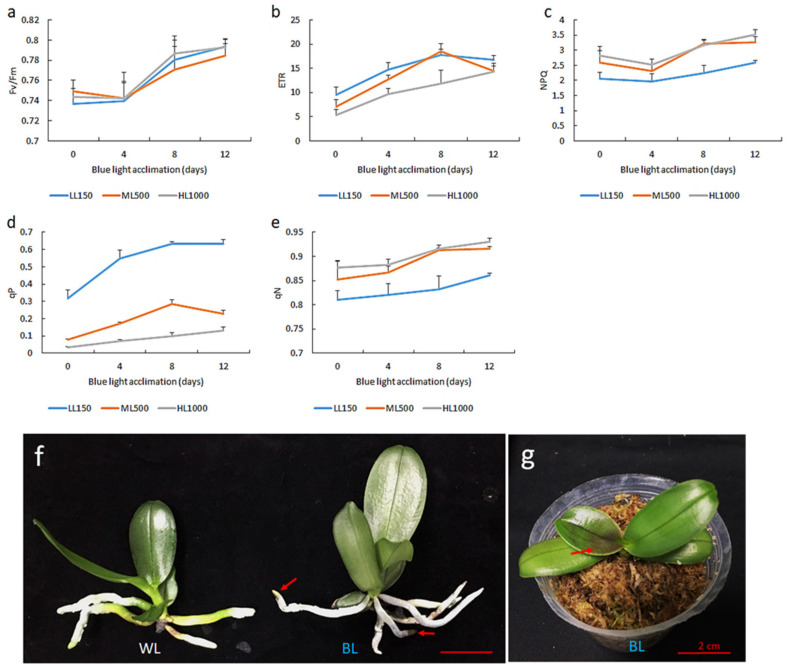
Time course of blue light acclimation to improve the chlorophyll fluorescence of *Phalaenopsis* seedlings. Young tissue culture seedlings were transplanted into a sphagnum moss 1.7” pot for a one-month establishment time and were then exposed to BL for 0, 4, 8 and 12 days, respectively. (**a**–**e**) The detection of chlorophyll fluorescence parameters, including Fv/Fm, electron transport rate (ETR), non-photochemical quenching (NPQ), photochemical quenching of fluorescence (qP) and non-photochemical quenching of fluorescence (qN), was measured by setting a fluorescence leaf-chamber light intensity at 150 (LL), 500 (ML) and 1000μmol m^−2^ s^−1^(HL), respectively. After BL treatment at 100 µmol m^−2^ s^−1^ for 12 days, anthocyanin accumulated in the (**f**) root tip and (**g**) young leaves of the orchid. Red arrows indicate the sites of anthocyanin accumulation.

To study the effect of BL on the photoinhibition of the moth orchid, we treated young orchid seedlings with BL continuously at 100 µmol m^−2^ s^−1^ (BL) for 0, 4, 8 and 12 days, respectively. For each BL time course, we measured the chlorophyll fluorescence parameters by setting fluorescence leaf-chamber light intensities of 150, 500 and 1000 µmol m^−2^ s^−1^, respectively. In general, a prolonged BL treatment period tended to increase these fluorescence parameters, but there were some variations in the response curves at different light intensities (Figure 2a–e). For example, at a low light irradiation of 150 µmol m^−2^ s^−1^, the orchid leaves had a maximum qP, and a prolonged BL treatment further increased the qP level (Figure 2d). On the contrary, NPQ and pN values were lower at 150 µmol m^−2^ s^−1^ than those in high light regimes >500 µmol m^−2^ s^−1^ (Figure 2c,e). In a HL regime of 1000 µmol m^−2^ s^−1^, orchids after BL treatment tended to have a lower ETR level than those with <500 µmol m^−2^ s^−1^ (Figure 2b). Compared to the control without BL treatment, BL treatment for 12 days had the highest level of the PSII system, presumably due to BL acclimation in plants. Therefore we chose a BL treatment for 12 days as our photo-acclimation condition, and challenged orchids with moderate light stress (500 µmol m^−2^ s^−1^, ML) and high light stress (1000 µmol m^−2^ s^−1^, HL) for further study. After BL acclimation for 12 days, orchid seedlings showed a high anthocyanin accumulation in the newly developed orchid leaves and in the root tip (Figure 2f,g). It seems that long-term BL acclimation is beneficial for orchid growth.

### 2.3. Growth Reduction under High Light Stress, but Blue Light Acclimation Reduced the Damage of Young Orchid Seedlings

**Figure 3:**
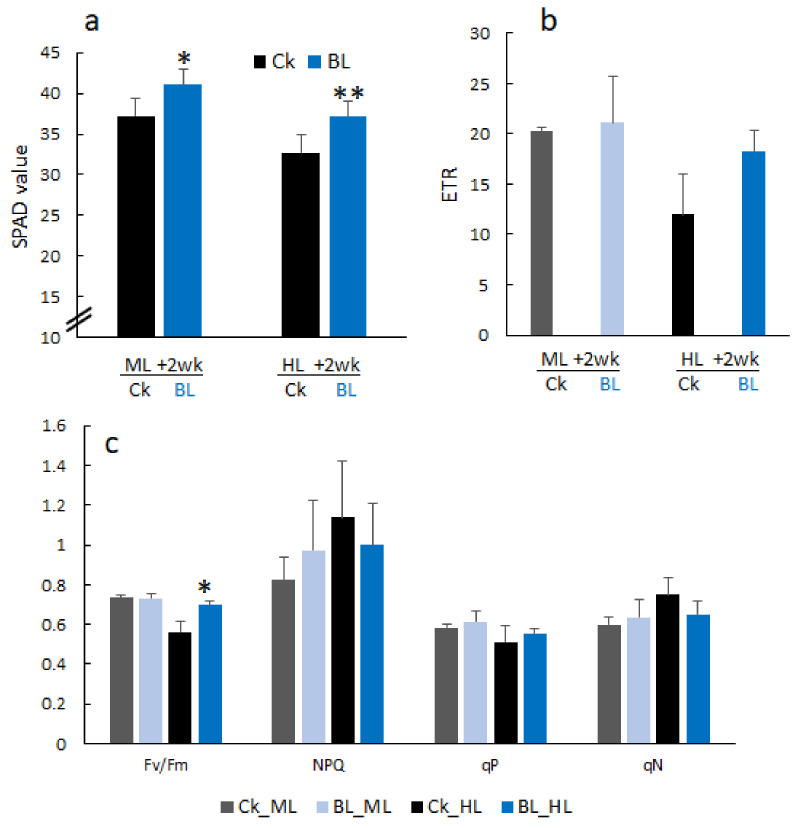
Photosynthesis of young orchid seedlings at two weeks after moderate and high light treatments, respectively. (**a**) SPAD value. (**b**) Electron transport rate (ETR). (**c**) Chlorophyll fluorescence parameters of Fv/Fm, non-photochemical quenching (NPQ), photochemical quenching of fluorescence (qP) and non-photochemical quenching of fluorescence (qN). ML, moderate light at 500 µmol m^−2^ s^−1^. HL, high light at 1000 µmol m^−2^ s^−1^. Significant differences in comparison with the control without BL acclimation (BL) are indicated with an asterisk. Statistical significance was determined by a one-way analysis of variance (ANOVA), followed by a Dunnett’s test. *, *p* < 0.05; **, *p* < 0.01. Error bars represent the standard error of the mean (*n* = 3).

**Figure 4:**
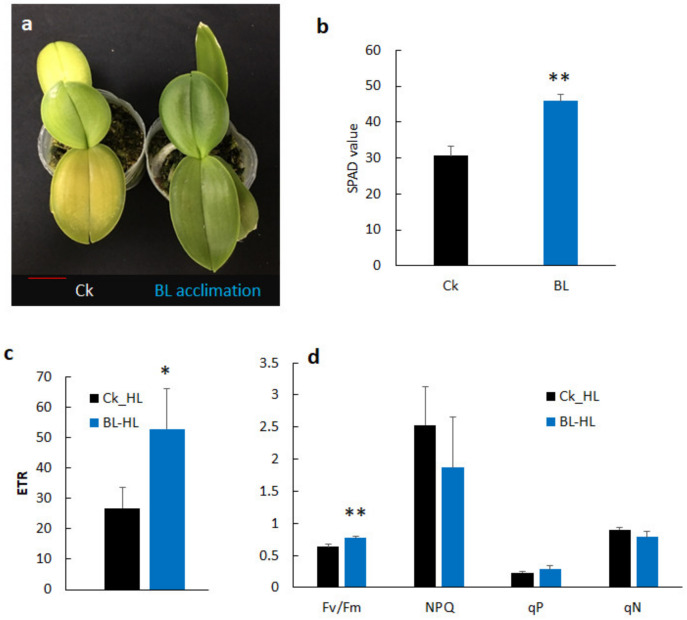
Blue light acclimation improved orchid growth and chlorophyll fluorescence. Orchid seedlings without (Ck) or with blue light acclimation for 12 days (BL) were grown under 1000 µmol m^−2^ s^−1^ high light conditions for 70 days. (**a**) Comparison of the growth of Ck and BL acclimation. Bar = 2 cm. (**b**) SPAD reading showed BL acclimation increased the chlorophyll content. Comparison of (**c**) ETR and (**d**) other chlorophyll fluorescence parameters between CK and BL acclimation. Abbreviations and statistical analyses are as described in the Figure 3 legend. *, *p* < 0.05; **, *p* < 0.01.

Orchid tissue culture young seedlings were BL-acclimated for 12 days (T0) before high light stress and were then transferred to ML and HL growth chambers, respectively. After two weeks of light stresses, those seedlings which were BL-acclimated had a higher SPAD reading and had a higher chlorophyll content compared to the control without BL acclimation (Figure 3a). Orchid seedlings irradiated with HL had a lower ETR compared to those in the ML condition. BL-acclimated orchids tended to acquire a slightly higher ETR and had a higher Fv/Fm than the control after HL exposure for two weeks (Figure 3b,c). Moreover, under HL stress conditions, BL-acclimated orchids still maintained higher levels of Fv/Fm compared to the control. After prolonged exposure under HL conditions for 70 days, young orchid seedlings without BL acclimation showed yellowish leaves due to photo-bleaching, but BL acclimation seedlings retained more healthy green leaves (Figure 4a, right panel), had a higher chlorophyll content and ETR (Figure 4b,c), and an increased Fv/Fm when compared with the control under severe HL conditions for 70 days (Figure 4d).

### 2.4. Growth Reduction under High Light Stress, but Blue Light Acclimation Reduced the Damage to 2.5” Potted Plants

**Figure 5:**
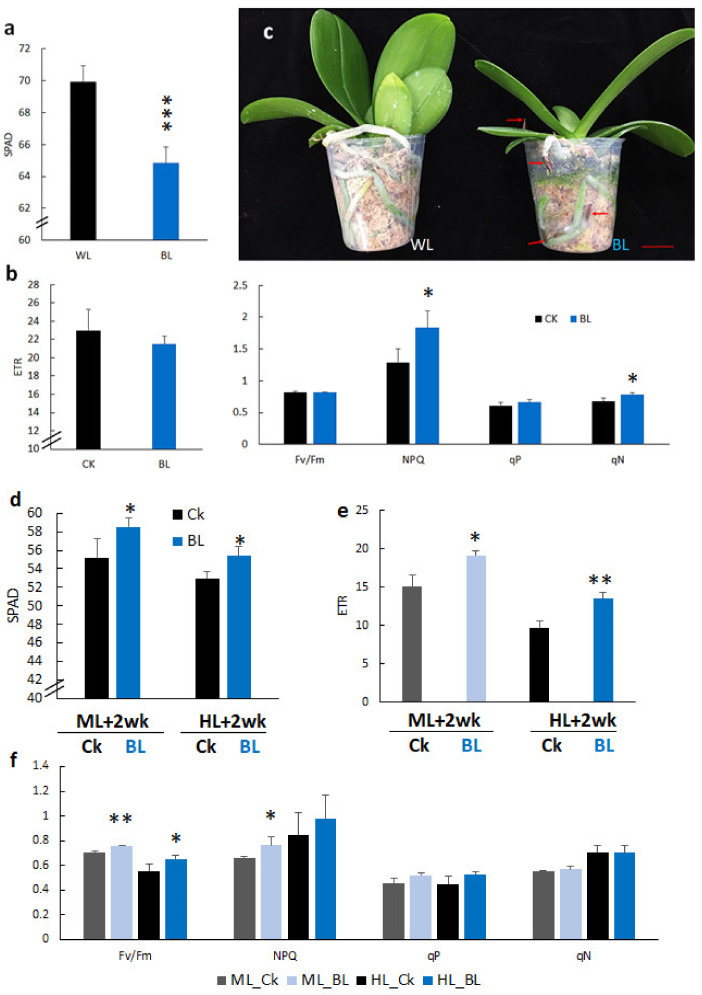
Blue light acclimation altered the growth and chlorophyll fluorescence parameters of orchid plants. (**a**) M1663 2.5” potted orchids treated with 100 μmol m^−2^ s^−1^ blue light for 12 days (T0) altered the SPAD value. (**b**) BL acclimation affected the chlorophyll fluorescence parameters. (**c**) The phenotype of orchid plants without or with BL acclimation for 12 days. Red arrows indicate the accumulation of anthocyanin in the orchid root tip. (**d**–**f**) Effect of BL acclimation-altered chlorophyll fluorescence of orchid plants after two weeks of exposure to moderate high (ML) and high light (HL) conditions. (**d**) SPAD value of orchid plants after two weeks of exposure to ML and HL. (**e**) ETR of orchid plants after being exposed to ML and HL for two weeks. (**f**) Chlorophyll fluorescence parameters of Fv/Fm, NPQ, qP and qN of orchid plants after being exposed to ML and HL for two weeks. ML, moderate light at 500 µmol m^2^ s^−1^. HL, high light at 1000 µmol m^2^ s^−1^. Abbreviations and statistical analyses are as described in the Figure 3 legend. *, *p* < 0.05; **, *p* < 0.01; ***, *p* < 0.001.

The 2.5” orchid potted plants that had been BL-acclimated for 12 days tended to have a lower SPAD reading than the control (Figure 5a). This may be due to the chloroplast avoidance effect of BL. Moreover, BL acclimation tended to increase the level of NPQ of orchid plants over that of the control without BL acclimation (Figure 5b). Anthocyanin was accumulated in the root tip of orchid plants after BL acclimation (Figure 5c, red arrows). Then, orchid plants with or without BL accumulation were exposed to ML or HL conditions, respectively, for two weeks. It was found that BL-acclimated plants had a higher chlorophyll content, ETR, Fv/Fm and NPQ than the control without BL acclimation (Figure 5d–f).

### 2.5. Effect of Blue Light Acclimation on Antioxidative Enzymes

**Figure 6:**
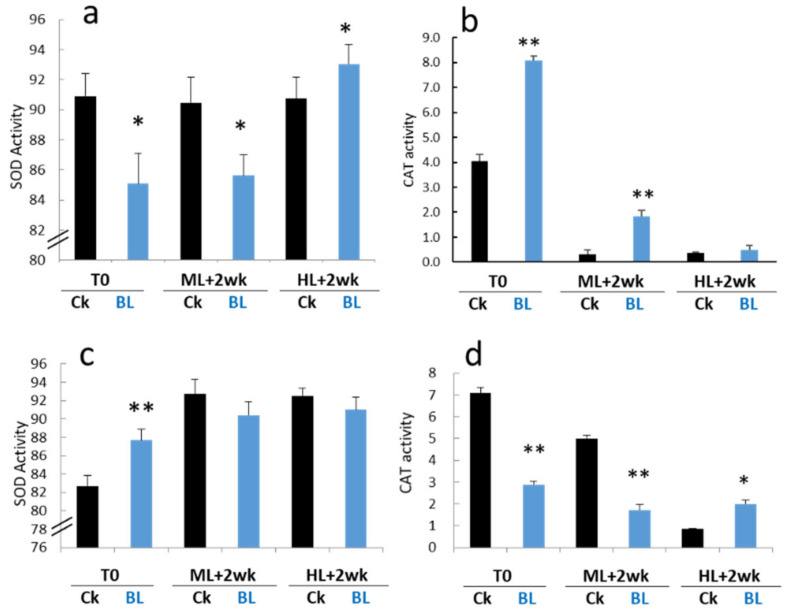
Blue light acclimation affects the antioxidant enzyme activities of moth orchids. (**a**,**b**) Young orchid seedlings, (**c**,**d**) mature 2.5” potted plants. (**a**,**c**) Superoxide dismutase (SOD) activities, (**b**,**d**) catalase (CAT) activity. CAT, Catalase activity (mU/mL). Statistical analyses are as described in the Figure 3 legend. Error bars represent the standard error of the mean (*n* = 3). *, *p* < 0.05; **, *p* < 0.01.

Some key enzymes that decrease ROS toxicity, such as SOD and CAT enzyme activities, were detected in the young leaves and mature leaves just after BL acclimation for 12 days (T0), and after exposure to ML or HL for two weeks, respectively. BL acclimation tended to reduce the SOD activities of young seedlings at T0 and ML, but it was increased at HL (Figure 6a). It was found that a high irradiation significantly inhibited CAT activity in young leaves, but BL-acclimated orchids retained a higher CAT than those controls without BL acclimation at T0 and ML (Figure 6b). For mature leaves, BL acclimation tended to increase SOD activity at T0 (Figure 6c). In contrast, BL-acclimated plants had a significantly reduced CAT activity compared to the control at T0 and after two weeks exposure to ML conditions. However, after two weeks of exposure to HL, BL-acclimated plants had a higher CAT activity than the control (Figure 6d). These results indicated that HL stress increased the cellular oxidating environment in orchids but that BL acclimation altered the antioxidant metabolism during prolonged HL exposure.

### 2.6. Blue Light Acclimation Altered Sugar Content in Moth Orchid

**Figure 7:**
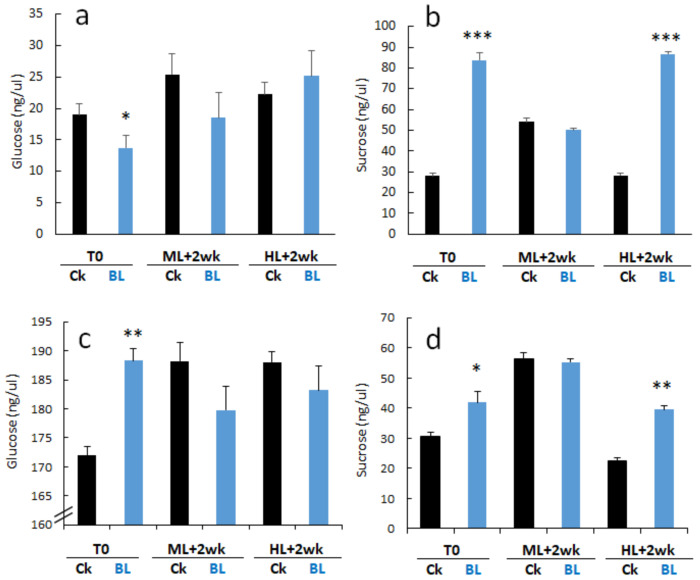
Blue light acclimation altered glucose and sucrose accumulation in moth orchids. (**a**,**b**) Young seedlings, (**c**,**d**) mature 2.5” potted plants. (**a**,**c**) Glucose content, (**b**,**d**) sucrose content. Statistical analyses are as described in the Figure 3 legend. Error bars represent the standard error of the mean (*n* = 3). *, *p* < 0.05; **, *p* < 0.01; ***, *p* < 0.001.

Glucose is the final product of photosynthesis, and sucrose is the major transported sugar in plants. Therefore, we determined the glucose and sucrose contents in young seedlings and orchid plants. The results indicated that orchid young leaves accumulated more sucrose than glucose. In contrast, mature leaves contained more glucose than sucrose. After BL acclimation, young leaves had a reduced glucose content at T0 (Figure 7a); they, in turn, accumulated more sucrose at T0 and after HL stress when compared to the control (Figure 7b). For mature leaves, BL acclimation significantly increased the glucose content at T0 (Figure 7c) and significantly increased the sucrose content in orchid plants at T0 and after HL stress.

### 2.7. Blue Light Acclimation Altered Gene Expression Patterns in Young Orchid Seedlings

**Figure 8:**
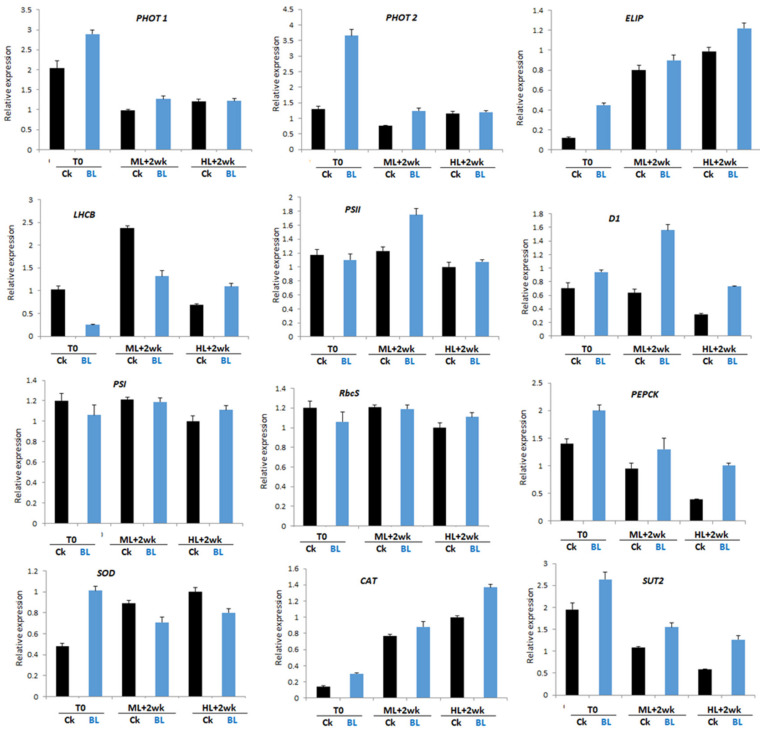
Quantitative RT-PCR shows that blue light treatment alters the gene expression patterns of young orchid seedlings. RNA samples were collected from young seedlings at two weeks after exposure to moderate light at 500 µmol m^2^ s^−1^ (ML) or high light at 1000 µmol m^2^ s^−1^ (HL). Error bars indicate the SD of the mean from three bioreplicates.

We collected leaf samples of orchid seedlings with and without BL acclimation for 12 days (T0) and irradiation at ML and HL for two weeks, isolated RNAs and performed qRT-PCR. The results indicated that, at T0, BL acclimation tended to increase the transcript levels of *phot1*, *phot2*, *ELIP*, *D1, PEPCK*, *SOD*, *CAT* and *SUT2* (Figure 8). In those seedlings that had been BL-acclimated and then exposed to a moderate light irradiation (ML) for two weeks, *phot1*, *phot2*, *PSII*, *D1*, *PEPCK*, CAT and *SUT2* were upregulated when compared to the control. With the exception of the genes associated with antioxidative enzymes, such as *SOD* and *CAT*, the orchid seedlings exposed to HL stress tended to have lower gene expression levels than those exposed to ML in most of the tested marker genes. Meanwhile, under HL stress conditions, BL-acclimated seedlings seemed to protect orchid growth by increasing the gene expression levels of *ELIP*, *LHCB*, *PSI*, *D1*, *RbcS*, *PEPCK*, *CAT* and *SUT2* (Figure 8).

### 2.8. Blue Light Acclimation Altered Gene Expression Patterns in Orchid 2.5” Potted Plants

**Figure 9:**
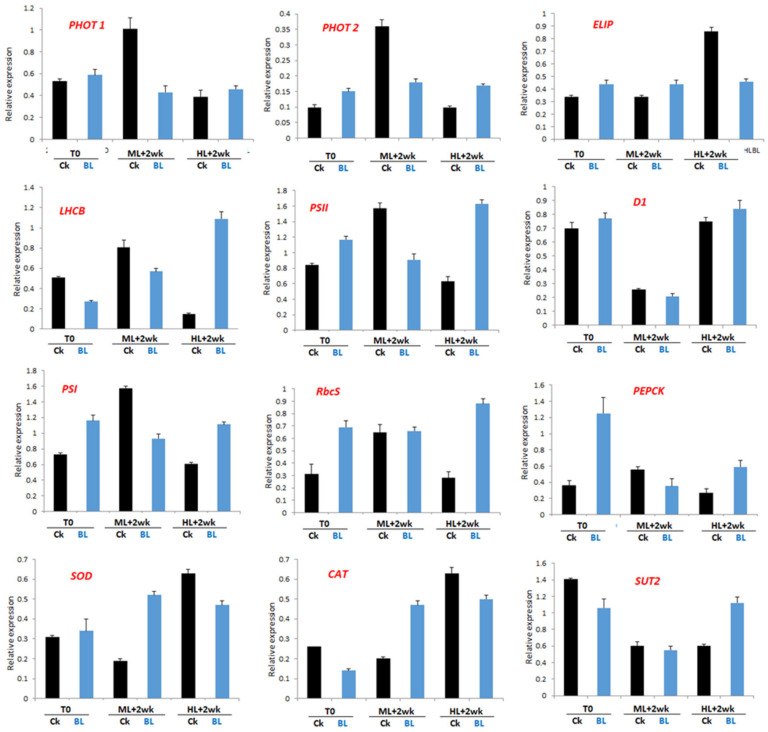
Quantitative RT-PCR showed that blue light treatment altered the gene expression patterns of mature orchid plants. RNA samples were collected from 2.5” potted plants at two weeks after exposure to moderate light at 500 µmol m^2^ s^−1^ (ML) or high light at 1000 µmol m^2^ s^−1^ (HL). Error bars indicate the SD of the mean from three bioreplicates.

We also collected mature leaf samples of orchid plants with and without BL acclimation for 12 days (T0) and after irradiation at ML and HL for two weeks, isolated RNAs and performed qRT-PCR. The results indicated that, after BL acclimation for 12 days (T0), orchid plants tended to increase *phot1*, *phot2*, *ELIP*, *PSII*, *D1*, *PSI*, *RbcS* and *PEPCK* transcript levels but decreased *LHCB*, *CAT* and *SUT2* mRNAs. After ML irradiation for two weeks, BL-acclimated plants upregulated *ELIP*, *SOD* and *CAT*, but decreased the *phot1*, *phot2*, *LHCB*, *PSII*, *PSI* and *PEPCK* gene expression. However, after HL irradiation for two weeks, BL-acclimated orchid plants tended to upregulate *phot1*, *phot2*, *LHCB*, *PSII*, *D1*, *PSI*, *RbcS*, *PEPCK*, *SOD*, *CAT* and *SUT2,* but decreased the *ELIP*, *SOD* and *CAT* gene expression levels when compared to those plants without BL acclimation control (Ck) (Figure 9).

## 3. Discussion

### 3.1. BL100 Induces Photo-Acclimation in Moth Orchid

Photoinhibition occurs when plants are exposed to excessive irradiation above saturation for photosynthesis. It has been reported that BL could induce photo-acclimation and thus assist plants’ tolerance to high light stress [15,16,17]. We also found that BL100 upregulated phototropins and significantly induced chloroplast avoidance in moth orchids [7]. This study further indicated that BL100 treatment for 12 days (T0) enhanced photo-acclimation in moth orchids. Anthocyanin accumulated in the newly developed orchid leaves and in the root tips (Figure 2f,g). It is reported that when plants are exposed to stressful conditions they typically accumulate anthocyanin to change the reflection and absorption of light penetration to leaf tissues. Besides that, anthocyanin has a photoprotective effect on the PSII [27]. It has been reported that Arabidopsis confers a better antioxidant potential and tolerance to high irradiation stress by increasing the anthocyanin content [28]. The moth orchid is highly sensitive to high irradiation because whenever the light intensity is above 300 μmol m^−2^ s^−1^, the ETR and qP levels were significantly reduced (Figure 1), indicating damage to PSII. However, BL acclimation assists orchids in acquiring a photo-acclimation state, with significant increases in the chlorophyll fluorescence levels of Fv/Fm, ETR, qP, NPQ and qN after 12 days of BL100 acclimation (Figure 2). Moreover, BL acclimation upregulated marker genes such as *ELIP*, *PSII* and *D1* in young (Figure 8) and mature leaves (Figure 9). These results suggest that BL acclimation mainly provides protection to the PSII complex of orchids. We observed that *PSI* mRNA was not much altered in the young leaves after BL acclimation but that it was upregulated in the mature leaves after BL treatment for 12 days (Figure 8 and Figure 9). Our data indicated that young orchid seedlings are more sensitive to photoinhibition than mature plants. That may be because the mature leaves have a more complete BL-acclimation potential than the young leaves. This finding coincides with another study on maize that indicates that a blue-light receptor operated more powerfully in mature leaves [29]. Furthermore, researchers compared cucumber grown under red and blue light (at the same intensity: 300 μmol m^−2^ s^−1^) and found that plants grown under BL conditions showed a normal photosystems activity, increased the ETR capacity and obtained a higher biomass, mainly because BL had better PSII and PSI activities [30].

### 3.2. BL100 Induces Chloroplast Avoidance and Enhances Chlorophyll Fluorescence

Orchid leaves significantly reduced the SPAD value after BL acclimation, showing that BL induced chloroplast avoidance (Figure 5a), the first photoprotection strategy to reduce the antenna size and decrease photodamage in orchids. This study showed that the gene expression levels of *phot 1* and *phot2* were significantly upregulated after BL acclimation for 12 days (Figure 8 and Figure 9), indicating that they may have contributed to the chloroplast avoidance. Under high irradiation, although BL-acclimated young seedlings have *phot1* and *phot2* that is upregulated more than twice as much as those in mature orchid plants, the young seedlings still have severe photo-bleaching after prolonged HL exposure (Figure 4). That may be because the young seedlings have a low PSII adjustment ability (Figure 3), leading to more severe photodamage after prolonged high light stress (Figure 4a). In contrast, mature plants are more able to tolerate HL conditions. BL-acclimated orchid plants are able to dissipate this energy via non-photochemical quenching mechanisms by increasing the NPQ (Figure 5f) and allowing orchid plants to tolerate high light intensities to avoid being damaged. A higher level of Fv/Fm and ETR, as shown in Figure 5, implies that BL acclimation has a better PSII activity under high irradiation. Consequently, after long-term high irradiation, BL-acclimated orchids have a higher chlorophyll content (Figure 5d) and higher levels of chlorophyll fluorescence than the control without BL-acclimation (Figure 5e,f).

Early light-inducible proteins (ELIPs) are chloroplast proteins related to the light-harvesting chlorophyll a/b gene family [20]. *ELIP* transcription is induced by BL, and its expression level is positively correlated with the degree of photoinhibition but decreases with the amount of D1 protein [31]. Our study showed that young orchid seedlings had an upregulated *ELIP* gene after exposure to HL stress (Figure 8); however, mature plants that were BL-acclimated and then exposed to HL had downregulated *ELIP* after exposure to HL stress (Figure 9).

### 3.3. BL Acclimation Induces Antioxidant Activity and Increases Sugar Content

ROS molecules might be induced by HL. Therefore, eliminating the toxic ROS molecules is essential for photoprotection. ROS scavenging systems, such as SOD, CAT, APX, GPX, etc., play essential roles in removing toxic ROS molecules in plant organelles [13]. This study detected enzyme activities of SOD and CAT and showed some contrasting results between young and mature leaves. Compared to the control without BL acclimation, BL-acclimated mature orchid leaves had an increased SOD activity after BL acclimation (Figure 6c). In contrast, a significant increase in CAT activity was found in the young leaves after 12 days of BL acclimation (Figure 6b). The upregulation of the *SOD* and *CAT* gene expression was correlated with the enzyme activities (Figure 6, Figure 8 and Figure 9). ROS molecules will trigger/alter the expression of specific genes. For example, ^1^O_2_^*^ triggers programs related to the overexcitation of PSII, while O_2_^−^ and H_2_O_2_ might turn other groups of genes on in order to mitigate the electron pressure on the reducing side of PSI [32]. A recent study showed that BL-exposed rose flowers increased SOD, CAT, POD and APX enzyme activities, maintained the membrane stability, and increased the sucrose and glucose contents [33]. This study also found that sucrose and glucose were increased after BL acclimation and during exposure to high irradiation (Figure 7) and that they may contribute to orchid photoprotection.

### 3.4. Future Perspective

**Figure 10:**
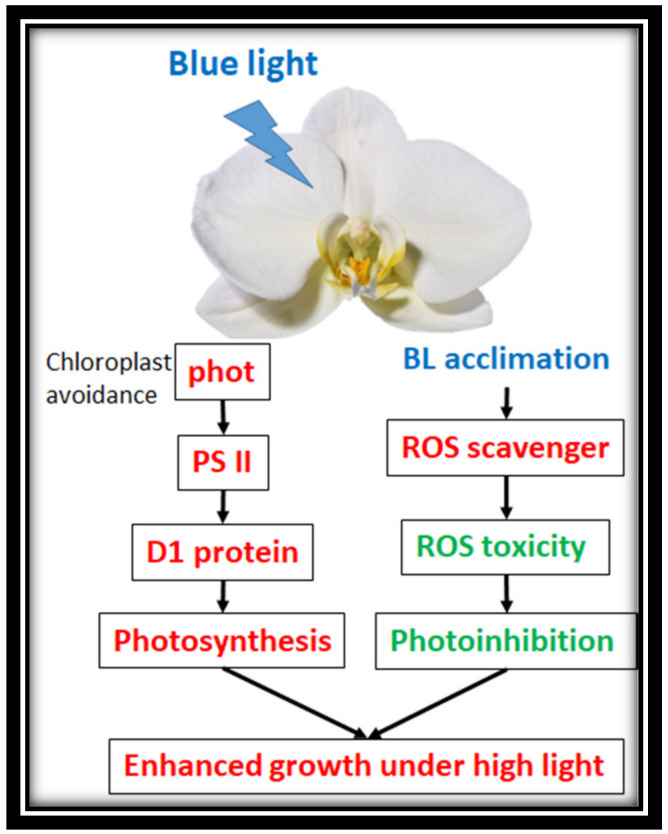
Outline of the findings of this study. This study showed that orchids pretreated with BL at 100 µmol m^2^ s^−1^ for 12 days had an enhanced chloroplast avoidance and acquired photo-acclimation, and increased gene expression levels of *PSII* and *D1 protein*, therefore increasing the photosynthesis rate. BL acclimation tended to increase antioxidant enzyme activities to remove the ROS toxic molecules and reduce photo-inhibition. Overall, BL acclimation enhanced photo-protection, reduced cellular damage and enhanced orchid growth under high irradiance conditions. Red fonts indicate the upregulation and additive effects on orchid growth. Green fonts indicate the downregulation and native effects on orchid growth.

Typically, moth orchids are planted in a greenhouse under one or two layers of black or silver color shade curtains to protect plants from photoinhibition. However, the fluctuation of sunlight in the greenhouse might still cause photodamage. This study showed that pretreatment with BL100 in young seedlings or mature plants confers photoprotection in both cases. Therefore, it is suggested to pretreat orchids (BL acclimation) before transplanting tissue culture young seedlings or transferring orchid plants to the greenhouse. This study discovered that BL-acclimatized orchids have several photoprotective advantages and molecular functions. For example, BL immediately turns the chloroplast avoidance mechanism on in order to reduce the antenna size, increase ETR and decrease light energy absorption. Figure 10 shows an outline of the findings of this study. In short, BL acclimation upregulates *phot1* and *phot2* gene expression and induces chloroplast avoidance, increases the gene expression of *PSII* and *D1*, enhances PSII repair, and maintains photosynthesis under HL. BL acclimation induces ROS scavengers and reduces ROS toxicity, therefore reducing the photoinhibition problem. Consequently, BL acclimation enhances orchid growth under high light stress.

A recent publication showed that transgenic plants overexpressing D1 protein have an increased photosynthesis efficiency and enhanced crop yield of *Arabidopsis*, tobacco and rice [34]. Moreover, overexpressing the RiskeFes protein (PetC), a component of the cyt b_6_f complex, also caused an increase in D1 and plant biomass, increased the photosynthesis efficiency of PSI and PSII, and led to an enhanced growth and crop yield [35]. This study shows that *D1* gene expression is upregulated by BL acclimation in orchids, presumably in association with an enhanced tolerance to high light damage. As the *Phalaenopsis* orchid is a shade-loving plant and is sensitive to high light, the question of how BL induced the D1 protein to repair PSII deserves further research in relation to this orchid. Understanding the redox stress of high light and antioxidant functions in orchids might assist us in manipulating greenhouse environments to enhance the photosynthesis and growth of *Phalaenopsis*. It has been claimed that the dynamic acclimation of photosynthesis enhances plant capability in response to stressful environments [36]. Obviously, there are complex coordinated interactions between the responses of photoreceptors, PSII and PSI, ROS, and metabolic responses to maintain photosynthesis under HL stress [37]. Knowledge gained from this study may help to further investigate and reduce the photodamage of orchids under high light stress and to enhance production.

## 4. Materials and Methods

### 4.1. Plant Material and Growth Conditions

*Phalaenopsis* orchid (*P. aphrodite* subsp. *Formosana*) cv. M1663 orchid tissue culture seedlings and 2.5” potted mature plants at the four-leaf stage (one year after transplanting to sphagnum moss) were purchased from Chain Port Orchid Nursery (Ping Tung, Taiwan). A total of 18 orchid plants were BL-acclimatized in a growth chamber set at a constant 26 °C, with continuous irradiation with LED blue light (BL, λ_max_ = 450 nm) at 100 μmol m^−2^ s^−1^, without a dark period in order to avoid other acclimations due to other factors such as darkness or a low temperature. After BL acclimation for 12 days (T0), three plants were measured for chlorophyll fluorescence, and three plants for destructive samples collection (RNA, ROS and biochemistry analysis). Then, six orchid plants were exposed to white light (WL) at 500 μmol m^−2^ s^−1^ (ML) or 1000 μmol m^−2^ s^−1^ (HL), respectively, for two weeks. Then, three plants were measured for chlorophyll fluorescence and three plants for destructive samples collection. The growth chambers were set at a constant 26 °C, with a 12 h light/12 h dark period. The control was kept in WL without BL acclimation and was then exposed to ML and HL, respectively.

### 4.2. SPAD Measurement

A Soil Plant Analysis Development (SPAD) chlorophyll meter (model SPAD-502) was used to measure the non-destructive chlorophyll content, as described previously [38]. In brief, the new fully expanded leaves (the first leaf, L1) were measured three times, and the data were averaged. Each treatment had four bioreplicates 

### 4.3. Measurement of Chlorophyll Fluorescence

Chlorophyll fluorescence was measured with a portable photosynthesis instrument LI-6800 (Li-Cor, Inc. Lincoln, NE, USA) equipped with a fluorescence leaf chamber 6800-01A (Li-Cor, Inc. Lincoln, NE, USA), with a 2 cm^2^ aperture for the fluorometer. The temperature and CO_2_ concentration were set, respectively, to 26 °C and 400 μmol mol^−1^. L1 leaves were dark-adapted for 2 h before measuring the parameters of chlorophyll fluorescence, and a saturating actinic light pulse of 8000 μmol m^−2^ s^−1^ was applied for 1 s to measure the maximal fluorescence. The actinic light was composed of 10% blue (470 nm) and 90% red (625 nm). Data of the Fv/Fm, electron transport rate (ETR), nonphotochemical quenching (NPQ), photochemical quenching of fluorescence (qP) and non-photochemical quenching of fluorescence (qN) were collected and presented. Each treatment had three bioreplicates.

### 4.4. Antioxidant Enzyme Activities

Leaf samples of the new fully expanded leaves (L1) of moth orchids with or without BL acclimation for 12 days (T0) and ML or HL exposure for two weeks were collected. A total of 100 mg of frozen leaf samples were homogenized. The SOD enzyme activity was determined using a Superoxide Dismutase (SOD) Activity Colorimetric Assay Kit (BioVision, Milpitas, CA, USA #K335-100) according to the instructions of the manufacturer. The SOD activity was calculated based on the percentage inhibition at 20 min at 37 °C. For the detection of the catalase enzyme activity, 100 mg of frozen leaf samples were homogenized and determined using a Catalase Activity Colorimetric/Fluorometric Assay Kit (BioVision, Milpitas, CA, USA #K773-100) according to the manufacturer’s instruction.

### 4.5. Measurement of Glucose and Sucrose

Leaf samples of the new fully expanded leaf (L1) of moth orchids with or without BL acclimation for 12 days (T0) and ML or HL exposure for two weeks were collected. A total of 100 mg of leaf samples were homogenized, 500 μL of 1×PBS buffer was added, and they were incubated for 30 min at 37 °C. Then, the glucose and sucrose concentrations were determined using a Glucose and Sucrose Colorimetric/Fluorometric assay kit (Sigma, Saint Louis, MS, USA #MAK013), following the instructions of the manufacturer.

### 4.6. Gene Expression Analysis

Moth orchids with or without BL acclimation for 12 days (T0) underwent ML or HL exposure for two weeks. Leaf samples of the new fully expanded leaves (L1) were collected. The total RNA was extracted and DNase-treated, and qRT-PCR was performed for 40 cycles with KAPA SYBR FAST qPCR Master Mix (2×) Kit (Kapa Biosystems, Woburn, MA, USA), as descripted previously [7]. The primers used in this study for PCR are listed in the Supplementary Table S1. The real-time RT-PCR reaction was performed in triplicate on the C1000^TM^ Thermal Cycler (Bio-Rad, Hercules, CA, USA). The relative gene expression level was normalized to *Ubiquitin* (PATC150470) as a control.

### 4.7. Statistical Analyses

Statistical analyses were determined by a one-way analysis of variance (ANOVA), followed by a Dunnett’s test. Probability values *p* < 0.05 were considered statistically significant.

## Supplementary Materials

Supplementary materials can be found at https://www.mdpi.com/1422-0067/21/17/6167/s1, Table S1: Primers used in this study.

## Abbreviations

BL	Blue light
ML	Moderate light
HL	High light
LHCB	Light-harvesting chlorophyll B-binding protein
RbcS	Ribulose bisphosphate carboxylase small subunit
ROS	Reactive oxygen species
SUT2	Sucrose transport protein
PSI	Photosystems I
PSII	Photosystems II
SOD	Superoxide dismutase
CAT	Catalase
