# Supplementary material for: Blue Light Acclimation Reduces the Photoinhibition of Phalaenopsis aphrodite (Moth Orchid)
Source: Int J Mol Sci. 2020 Aug 26;21(17):6167. doi: 10.3390/ijms21176167 (PMC7503704; doi:10.3390/ijms21176167)
Supplement: Supplementary file 1 [file ijms-21-06167-s001.pdf]

Table S1. Primers used in this study.

Primer Name	PATC No.	Sequence (5′ →3′)
PaUbi-F	PATC150470	TGAACTCCATCGCCTTCCTCTTC
PaUbi-R		TGAAGCATGGCATCAATTTC
PhoT1-qPCR-F	PATC143787	GCACCCTCACCACTCTTCGCCG
PhoT1-qPCR-R		AACTCCGATGCGACCCAGACTCCC
PhoT2-qPCR-F	PATC140643	GCCAGTGTCCTTAATCCGCTATGACG
PhoT2-qPCR-R		CATGTCATGCAGAGGAGTCCATCTTCC
PaELIP-F	PATC209823	AGCAAACATGCCGTCCTACAC
PaELIP-R		GGAGACGCAAGAGATGAATATGG
LHCB-qPCR-F	PATC141354	GCCAACTCCATTCCTCGGTCA
LHCB-qPCR-R		AGCAGTGTCCCAGCCGTAATCA
PSII-qPCR-F	PATC156580	CAAGACTGACACACCCTATGGACCT
PSII-qPCR-R		CCCTCCAAGTAGTCCAAGTAGTGTCA
Pa-D1-F	PAXXG225320	TATCATTGCCTTCATTGTTGCCC
Pa-D1-R		AAGTTCATAAGGACCGCCATTGTAC
PSI-qPCR-F	PATC154904	CCTCGTCGTCATCCTCACAATCT
PSI-qPCR-R		ACACCAGATATGCCTCCAAAGAAGAAAC
RBCS-ch-F	PATC155717	TGATGATCTCATCCGCTACCGC
RBCS-ch-R		CAGGGAGGTATGACAGTGTCTCAAAC
PEPCK-F	PATC138016	GCTTCCTACCCTATCGAGTACATTCC
PEPCK-R		TGGCTTGCGGCTCCTTGAT
Pa-SOD-F	PATC154840	GGCTGCTTGTCAACTGGTGCTC
Pa-SOD-R		GCGTGGACTACAACAGCCCTTC
Pa-CAT-F	PATC144989	GGATGATGAAGCTGTGATTGTTGG
Pa-CAT-R		CAGGCTGAAGAGGCAGGATGTC
PaSUT2-F	PATC148938	TACTCAACATTTCCATCGTCATCCC
PaSUT2-R		AGTTAGAGCGAGAGAGCCTTGGAA
